# GPU-accelerated MitoGraph for high-throughput three-dimensional mitochondrial morphology analysis

**DOI:** 10.1186/s12859-026-06441-z

**Published:** 2026-06-20

**Authors:** Siddharth Nahar, Zichen Wang, Eric Arkfeld, Gillian McMahon, Hiroyuki Hakozaki, Johannes Schöneberg

**Affiliations:** 1 Department of Pharmacology, University of California San Diego, Room 106B, La Jolla, San Diego, CA 92093 United States; 2 Department of Chemistry and Biochemistry, University of California San Diego, Room 106B, La Jolla, San Diego, CA 92093 United States

**Keywords:** Mitochondria, Fluorescence microscopy, Image processing, Image segmentation, Network analysis, GPU acceleration

## Abstract

MitoGraph is a widely used tool for the automated segmentation of mitochondrial networks in three-dimensional (3D) fluorescence microscopy. However, the emergence of advanced live-cell microscopes such as lattice light-sheet microscopy (LLSM) has produced massive four-dimensional (4D, 3D+time) datasets that highlight a critical bottleneck: current CPU-based implementations are computationally prohibitive, often requiring days or weeks to process. To address this limitation, we developed MitoGraph-GPU, a Python-based GPU implementation that accelerates the dominant filtering steps by vectorizing Hessian/eigenvalue and vesselness computations using CuPy, and streamlines network processing with faster skeletonization and topology analysis. Tested across budding yeast and human lung organoid datasets, MitoGraph-GPU achieves up to 11× speedup in yeast cells and 30× speedup in per-frame segmentation of lung cells. Segmentation fidelity is preserved, with  ~99.9% agreement in maximum intensity projections of segmented images, and minimal differences in downstream measurements. Critically, this throughput enables practical analysis of large 4D datasets : in an LLSM organoid use case (10 movies, 60 frames, ~ 50 cells per movie), total processing time decreases from ~ 500 h on CPU to ~ 20 h on GPU (25× faster). By producing accurate mitochondrial surfaces and skeletons, MitoGraph-GPU can serve as an efficient segmentation module for downstream mitochondrial tracking and analyses, enabling scalable high-throughput 4D mitochondrial phenotyping.

**Supplementary Information:**

The online version contains supplementary material available at https://doi.org/10.1186/s12859-026-06441-z.

## Background

Mitochondria form highly dynamic tubular networks [1] whose morphology and connectivity report on core cellular processes, including bioenergetic state [2], stress responses [3], and organelle quality control [4]. Quantifying these networks from fluorescence microscopy therefore remains a central need in cell biology, particularly as imaging modalities increasingly capture volumetric structure over time [5]. MitoGraph [6, 7] is a widely used tool for automated segmentation of tubular structures such as mitochondrial networks, originally developed for budding yeast and later extended to mammalian cells. While generalized frameworks have recently emerged to handle diverse organelles [8], MitoGraph remains relevant for mitochondrial research for its specialized mathematical tuning for tubular geometries. By utilizing a vesselness filter specifically optimized for thin, interconnected structures, it provides high-fidelity representations of organelle structure and connectivity. Furthermore, MitoGraph’s condensed graph-centric outputs provide a computationally efficient alternative to processed image data. These graph representations are essential for downstream graph analysis, as well as network tracking and temporal analysis tools [9]. MitoGraph continues to be used in mitochondrial research, for example recently in defining the local signals governing mtDNA purifying selection [10].

However, the scale of contemporary imaging has outpaced the practical throughput of the original MitoGraph implementation. Advanced approaches such as lattice light-sheet microscopy [11, 12] (LLSM) can generate very large four-dimensional (4D, space and time) datasets of subcellular structures (up to ~ 3 TB/hour), for which MitoGraph processing can require days to weeks using current CPU implementations. This computational latency limits experimental iteration, constrains sample sizes, and makes high-throughput 3D/4D analyses difficult to perform routinely. To address this gap, we have developed a Python-based, GPU-accelerated implementation of MitoGraph that substantially reduces runtime while maintaining segmentation accuracies. The new MitoGraph-GPU is ~ 11-fold faster on budding yeast datasets and ~ 30-fold faster on lung organoid datasets run on a standard desktop GPU, enabling scalable mitochondrial segmentation for large-scale mitochondrial processing.

## Implementation

MitoGraph segments and analyzes mitochondrial networks from 3D fluorescence microscopy images. The algorithm consists of three main steps: (1) segmentation of mitochondrial structures using derivative filters, (2) skeletonization to extract the centerline of the network, and (3) topological analysis to identify junctions and endpoints. Below, we describe the original implementation of MitoGraph and our GPU-accelerated approach.

### Segmentation using derivative filters

The segmentation stage of MitoGraph employs a vesselness filter followed by a divergence-based derivative filter to enhance mitochondrial structures and suppress background responses. Vesselness filtering is performed in a multi-scale manner on Gaussian-smoothed images, with the analysis conducted at the original image resolution to preserve fine mitochondrial structures. The scale range is fully tunable (σ_i, σ_f, dσ), and Gaussian smoothing uses the default CuPy truncation factor of 4, with the kernel radius defined as *round*(4σ) voxels, resulting in a kernel size of 2·*round*(4σ) + 1 voxels.

#### Vesselness filter

The first filter, the vesselness filter [13], leverages the eigenvalues of the Hessian matrix to detect tubular structures. The Hessian matrix is computed using Gaussian kernel convolutions, and the vesselness measurement is calculated across multiple scales to determine the optimal response for each voxel. A key modification in MitoGraph is the inclusion of a Frobenius norm threshold, which prevents unnecessary calculations outside regions of interest, ensuring computational efficiency. For a voxel * (x,y,z)* the Hessian matrix * H* is defined as:

$$ {\mathbf{H}} = \left[ {\begin{array}{*{20}c} {\frac{{\partial ^{2} F}}{{\partial x^{2} }}} & {\frac{{\partial ^{2} F}}{{\partial x\partial y}}} & {\frac{{\partial ^{2} F}}{{\partial x\partial z}}} \\ {\frac{{\partial ^{2} F}}{{\partial x\partial y}}} & {\frac{{\partial ^{2} F}}{{\partial y^{2} }}} & {\frac{{\partial ^{2} F}}{{\partial y\partial z}}} \\ {\frac{{\partial ^{2} F}}{{\partial x\partial z}}} & {\frac{{\partial ^{2} F}}{{\partial y\partial z}}} & {\frac{{\partial ^{2} F}}{{\partial z^{2} }}} \\ \end{array} } \right], $$

where  F=I*G is the convolution of the input image * I* with a Gaussian kernel * G*:

$$\:G(x,y,z)=\frac{1}{(2\pi\:{\sigma\:}^{2}{)}^{\frac{3}{2}}}exp\left(-\frac{{x}^{2}+{y}^{2}+{z}^{2}}{2{\sigma\:}^{2}}\right)$$

The eigenvalues $\:\left|{\lambda\:}_{1}\right|\le\:\left|{\lambda\:}_{2}\right|\le\:\left|{\lambda\:}_{3}\right|$ of * H* describe the local geometry of the voxel. The vesselness measurement * V* is computed as:

$${{V} = }\left\{ {\begin{array}{*{20}l} {0,} & {{\mathrm{if}}\,\lambda _{2} \, > \,0\,{\mathrm{or}}\,\lambda _{3} \, > \,0\,{\mathrm{or}}\,f\, < \,f_{T} ,} \\ {\left[ {1 - \exp \left( { - \frac{{R_{a}^{2} }}{{2\alpha ^{2} }}} \right)} \right]\exp \left( { - \frac{{R_{b}^{2} }}{{2\beta ^{2} }}} \right)\left[ {1 - \exp \left( { - \frac{{s^{2} }}{{2\gamma ^{2} }}} \right)} \right],} & {{\mathrm{otherwise,}}} \\ \end{array} } \right.$$

            

where $\:{R}_{a}=\frac{\left|{\lambda\:}_{2}\right|}{\left|{\lambda\:}_{3}\right|}$, $\:{R}_{b}=\frac{\left|{\lambda\:}_{1}\right|}{\sqrt{\left|{\lambda\:}_{2}{\lambda\:}_{3}\right|}}$, and $\:S=\sqrt{{\lambda\:}_{1}^{2}+{\lambda\:}_{2}^{2}+{\lambda\:}_{3}^{2}}$. The Frobenius norm * f* of the Hessian matrix is given by:

$$\:f\left(H\right)=\sqrt{{{\sum\:}_{i=1}}^{3}{{\sum\:}_{j=1}}^{3}H(i,j{)}^{2}}$$

A threshold $\:{f}_{T}=\sqrt{p\cdot\:{f}_{max}}$ is applied to exclude voxels outside the region of interest.

#### Divergence filter

The second filter, the divergence filter [14], computes the divergence of the normalized vesselness gradient field to counteract the blurring effect introduced by the Gaussian kernel. This filter emphasizes boundaries and prevents the merging of nearby structures, generating a divergence-stack that is subsequently thresholded to segment the mitochondrial network. To address the blurring effect of the Gaussian kernel, a divergence filter is applied to the vesselness stack. The divergence of the normalized vesselness gradient field is computed as:

$$\:D(x,y,z)=\nabla\:\cdot\:\left(\frac{\nabla\:V}{|\nabla\:V|}\right)$$

After applying the divergence filter, the resulting divergence-stack is processed to generate a binary segmented image. This is achieved by applying a post-divergence threshold $\:{D}_{c}$ (where $\:0\le\:{D}_{c}\le\:1$) to the divergence-stack. Voxels with divergence values lower than $\:{D}_{c}$ are set to zero, while those with values higher than or equal to $\:{D}_{c}$ are set to 255, effectively segmenting the mitochondrial network from the background. MitoGraph typically uses a default threshold of $\:{D}_{c}=\frac{1}{6}$, which has been observed to produce consistent and reliable results.

### Skeletonization and topological analysis

The segmented mitochondrial network is skeletonized to extract its centerline. The original MitoGraph implementation uses an iterative thinning algorithm to preserve the topology of the network. In our optimized python approach, we replaced this with the skeletonization routine from the scikit-image library [15], which achieves faster performance while maintaining topological integrity.

For topological analysis, we used the python-igraph library [16] to manipulate and analyze the skeletonized network. Junction points and endpoints were identified by analyzing the degree of each voxel in the skeletonized network using list-based manipulations.

### Optimized implementation

We re-implemented the MitoGraph algorithm in Python, leveraging GPU acceleration and optimized libraries to improve computational efficiency. Key modifications include:

1. *GPU-Accelerated Filtering*: The computation of the Hessian matrix, eigenvalues, and vesselness measurements was vectorized and implemented using CuPy [17], a GPU-accelerated library for numerical computations. Convolutions with the Gaussian kernel were performed using CuPy’s FFT-based convolution, significantly reducing computation time.
2. *Efficient Skeletonization*: The iterative thinning algorithm was replaced with the skeletonization routine from the scikit-image library, which preserves topology while achieving faster performance.
3. *Topological Analysis*: Junction points and endpoints were identified using the python-igraph, enabling efficient analysis of the skeletonized network. We also utilize CUDA [18] kernel for point wise intensity and smoothing calculations.

To evaluate the performance and accuracy of our new implementation, we applied MitoGraph-GPU to two types of mitochondria fluorescence datasets:

#### Budding yeast dataset

The budding yeast dataset was obtained from the MitoGraph repository (https://github.com/vianamp/MitoGraphTools/). This dataset comprises five images of mitochondria in budding yeast cells cultured in glucose-rich media. The images were acquired using a spinning disk confocal microscope, with voxel size of 0.056 × 0.056 × 0.200 μm (xyz) in 16-bit integer.

#### Human lung organoid dataset

Branching lung organoids were grown from human induced pluripotent stem cells with fluorescent tags on the mitochondria (TOM20-GFP) and cell membrane (CAAX-RFP). Day 68 organoids were imaged with LLSM. The LLSM data was preprocessed using the LiveLattice pipeline [19]. The preprocessed data has isotropic voxel size of 0.111 × 0.111 × 0.111 μm (xyz) in 16-bit integer. Single-cell mitochondria 4D movies were extracted using the cell membrane channel as the mask.

All CPU implementations are run on an AMD Ryzen Threadripper PRO 3955WX CPU and all GPU implementations are run on a NVIDIA RTX 3090 Ti GPU.

## Results

### MitoGraph-GPU drastically improves the throughput of mitochondrial segmentation

**Fig. 1:**
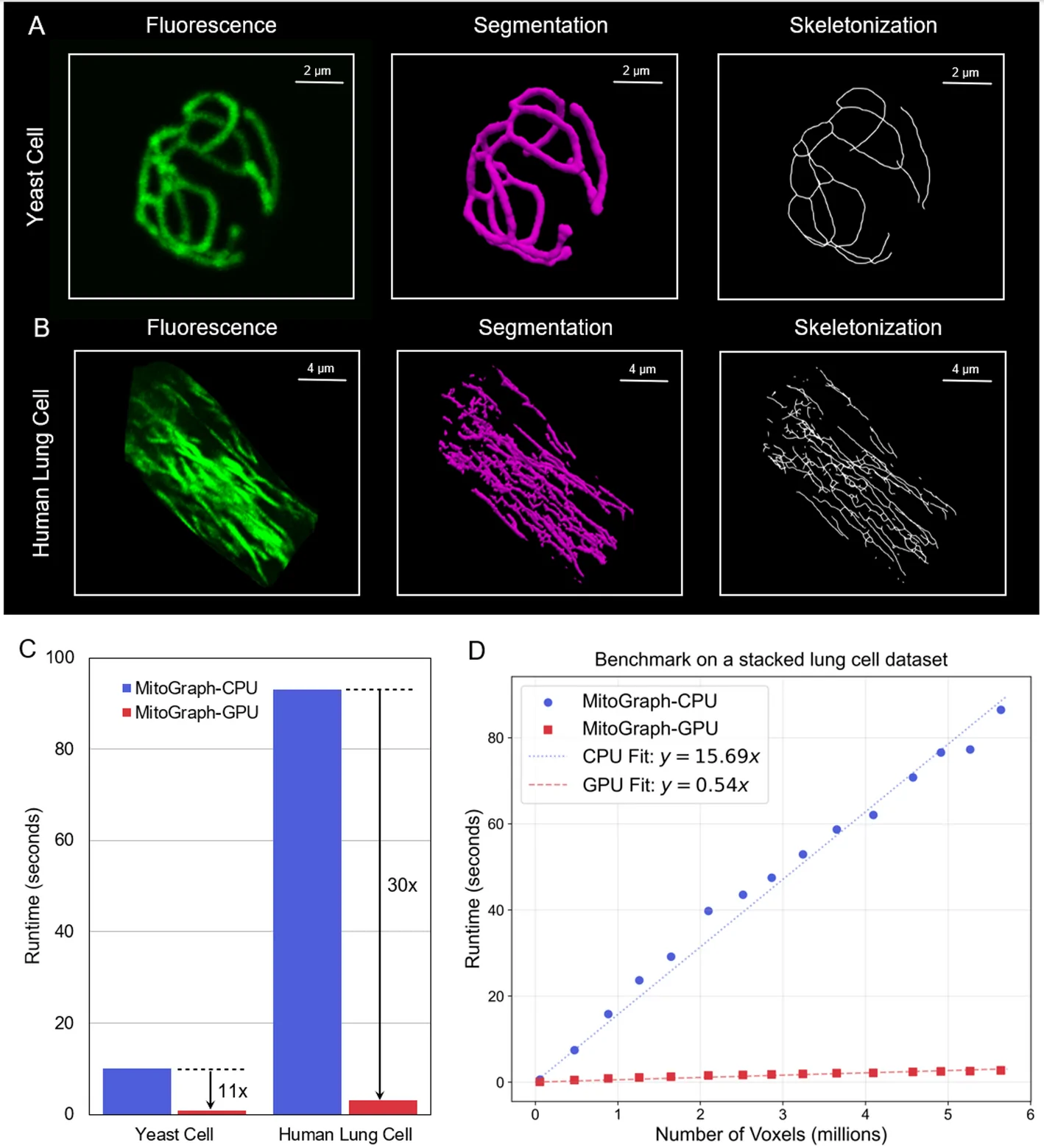
GPU-acceleration drastically reduces the runtime of segmentation by MitoGraph. MitoGraph inputs the 3D fluorescence images (top left), and outputs the segmented surfaces (top middle) and skeleton (top right), with examples in (**A**) yeast cell and (**B**) human lung cell. We used napari [20] for the visualization of 3D fluorescence images and ParaView [21] for rendering surface and skeleton VTK files. (**C**) MitoGraph runtime is reduced from 10 s to 0.9 s (11x) for yeast cell dataset and from 93 s to 3.1 s (30x) for lung cell dataset through GPU acceleration. (**D**) Runtime benchmarks on a large volume built from stacking lung cell volumes show that both the CPU (blue) and GPU (red) versions scale linearly with crop size. The GPU-accelerated version consistently achieves a ~ 30-fold speedup over the CPU baseline (linear fits shown) in this benchmark dataset

MitoGraph uses the raw fluorescence image as the input to segment the mitochondria surface and extract mitochondrial skeleton. Our GPU-accelerated implementation achieves an 11× speedup for the budding yeast dataset (Fig. 1A) and up to a 30× speedup for the human lung cell dataset (Fig. 1B). The disparity in speedup between the yeast and organoid datasets is primarily due to differences in total image volume and mitochondrial density. Smaller yeast datasets contain fewer voxels, leading to underutilized GPU resources where fixed costs like kernel launch overhead become disproportionately significant. Conversely, the denser mitochondrial networks in organoids provide the higher computational load and parallelism required to fully saturate the GPU, resulting in superior performance gains.

We conducted experiments to assess the scalability of MitoGraph and our optimized MitoGraph-GPU with varying data sizes (in number of voxels). We first tiled a base lung cell volume (140 × 140 × 100 voxels, xyz) into a 3 × 3 × 3 grid to form a large volume (420 × 420 × 300 voxels, xyz). We then evaluated performance by sampling voxels at varying crop sizes. The resulting runtimes for the CPU (blue) and GPU-accelerated (red) implementations were plotted against the total number of voxels per crop. Both implementations exhibited linear scaling, as confirmed by linear fitted lines, as well as the log-log axis plot with slope = 1 (Supplementary Fig. 1). The GPU-accelerated version consistently outperformed the CPU implementation by approximately 30-fold across all tested crop sizes. Notably, the GPU implementation also exhibits a 5-fold improvements over acceleration through NumPy-based vectorization alone (Supplementary Fig. 2). This improvement highlights the scalability of the software, making it highly efficient for processing large-scale datasets.

### MitoGraph-GPU accurately reproduces the segmented mitochondrial structure

**Fig. 2:**
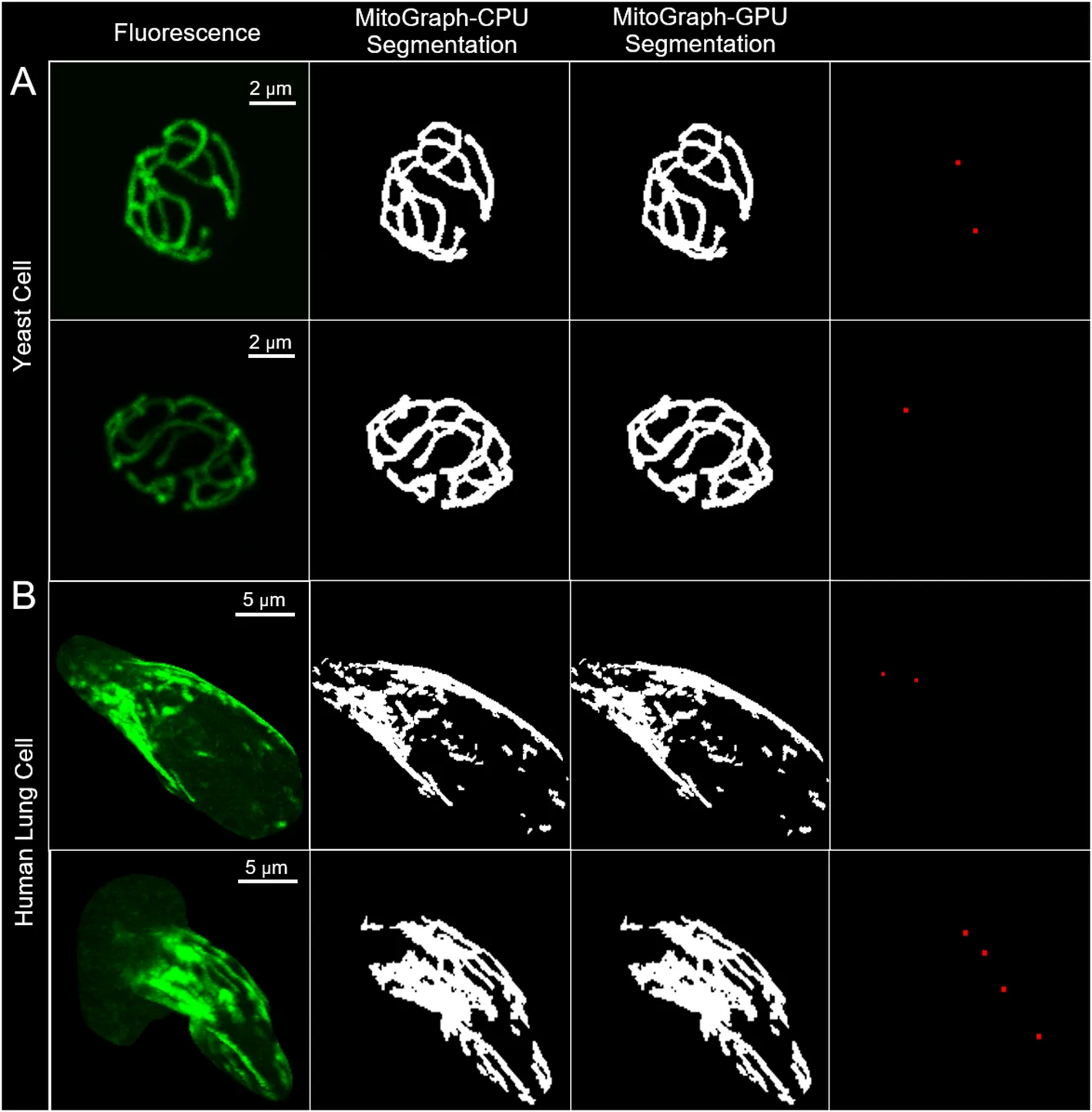
GPU implementation shows minimal differences in the maximum intensity projections of the segmented binary images compared to the original MitoGraph. Per-pixel differences for the maximum intensity projection of the two approaches are shown as red dots for (**A**) yeast cells and (**B**) lung cells. The pixel difference is 0.025% yeast cells and 0.16% lung cells

In our optimized implementation, we vectorized both filters and employed CuPy-accelerated methods for convolutions and labeled comprehensions, enhancing computational efficiency. To validate the correctness of our approach, we compared the segmented outputs from the original algorithm and our GPU-accelerated version by examining the pixel differences in the maximum intensity projections of the segmented images (Fig. 2A-B). Our GPU-accelerated method maintains high accuracy (~ 99.9%) with minimal deviation from the original outputs, ensuring reliable segmentation while achieving significant speed-up.

The main source of variation comes from the skeletonization step. The C++ VTK pipeline in the original MitoGraph uses a custom thinning strategy that iteratively removes boundary voxels according to a direction-dependent template-matching procedure, maintaining explicit surface and deletion lists throughout. In contrast, our GPU implementation employs the Lee et al. algorithm, which generates a medial axis transform followed by distance-ordered homotopic thinning to preserve topology. These fundamentally different approaches influence both computational efficiency and the morphology of the resulting skeleton, leading to minor structural variations between outputs.

**Fig. 3:**
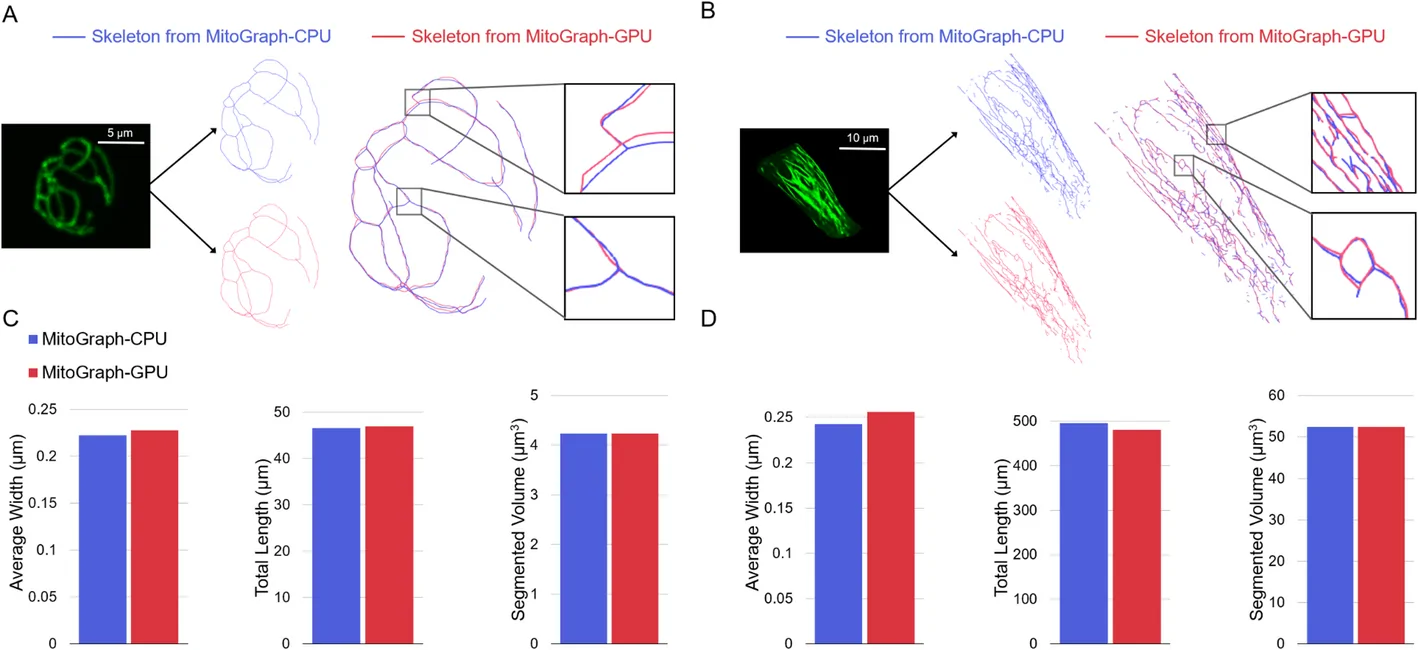
GPU implementation shows minimal differences in the mitochondrial skeleton and measured features compared to the original MitoGraph. The mitochondrial skeleton from the original CPU version (blue) is overlaid onto that from the new GPU version (red) for (**A**) yeast cells and (**B**) human lung cells. Network features including average tubular width, total skeleton length and segmented volume are compared between the two versions for (**C**) yeast cells and (**D**) human lung cells

After skeletonization, we extracted junctions and endpoints by identifying voxels with degrees other than 2, representing bifurcations and termini. Additionally, we computed quantifiable measurements such as average tubular width, total skeleton length, and mitochondrial volume (from segmented voxels). The overall average difference (mean ± std) between the quantifiable measurements for MitoGraph-CPU and MitoGraph-GPU across the dataset is less than 6% (Table 1). Comparing the two implementations across yeast and human lung cells, the superimposed mitochondrial skeletons demonstrate excellent co-localization and consistent topological structures (Fig. 3A-B). The three quantifications across both test cases indicate minimal variation between the two implementations (Fig. 3C-D).

**Table 1:** The average variation (mean ± std) in network quantifications between MitoGraph-CPU and MitoGraph-GPU across both datasets

Dataset	Average width (μm)	Total length (μm)	Volume (# voxels)
Budding Yeast	1.795 (± 0.993)%	1.653 (± 0.837)%	0.0356 (± 0.023)%
Lung Organoid	5.341 (± 0.550)%	2.353 (± 1.444)%	0.0253(± 0.017)%

### MitoGraph-GPU enables efficient analyses 

**Fig. 4:**
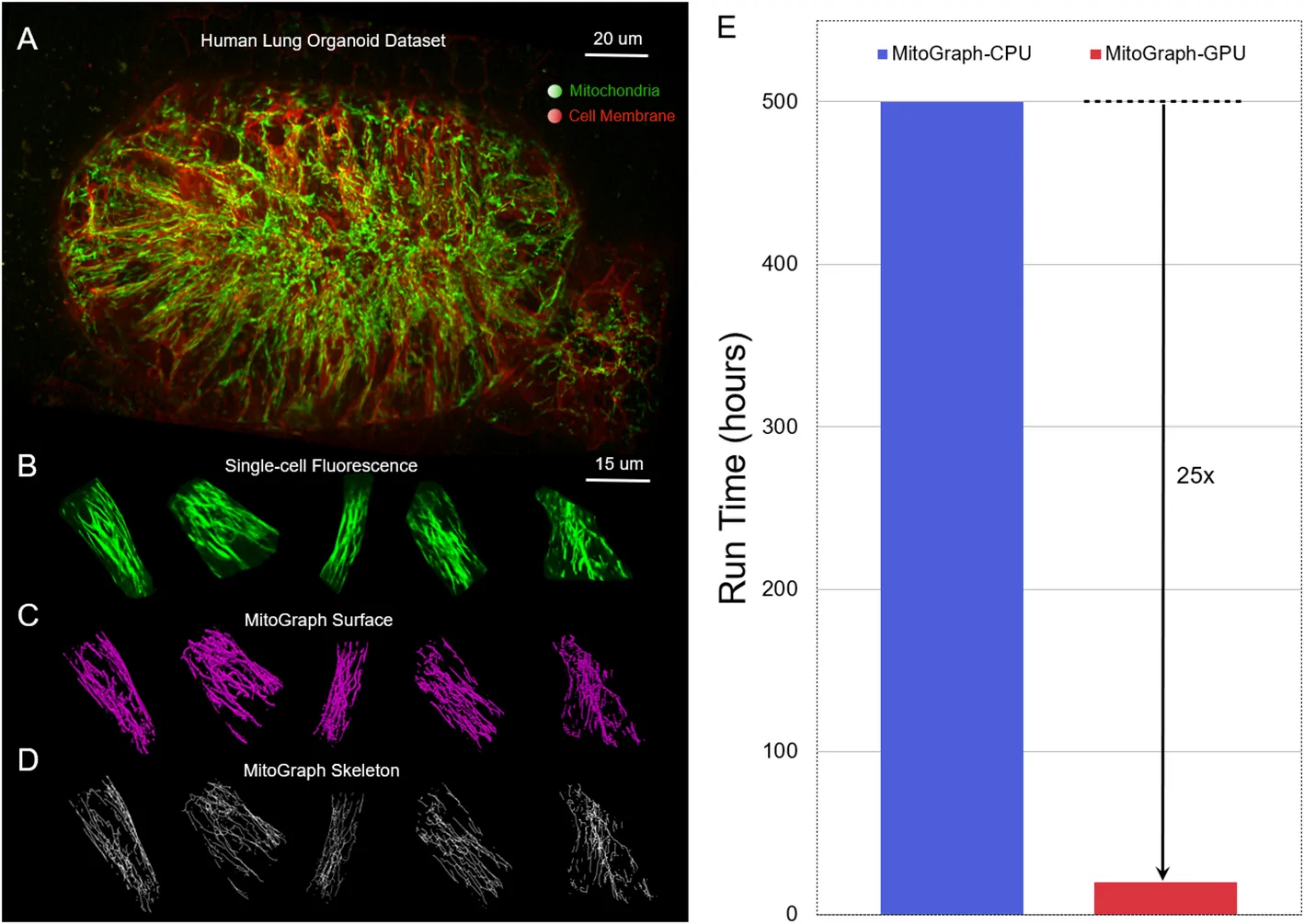
GPU implementation allows efficient mitochondrial segmentation for massive 4D LLSM dataset. (**A**) LLSM 4D dataset of mitochondria (green) and plasma membrane (red) in human lung organoids. (**B**) Single cells can be extracted based on the membrane channel. (**C**-**D**) MitoGraph uses single-cell fluorescence images to extract mitochondrial surface and skeleton. (**E**) Processing 500 60-frame single-cell movies using MitoGraph-CPU requires ~ 500 h of computation while MitoGraph-GPU requires only ~ 20 h, representing a 25-fold speed-up

Due to the drastically speed-up implementation of MitoGraph, it is now possible to analyze massive 4D mitochondrial datasets within a reasonable timeframe. As an example use case, our human lung organoid datasets includes 10 volumetric time-lapse movies (108 × 120 × 30 μm, 60 frames), each containing approximately 50 cells (Fig. 4A). With the MitoGraph-CPU implementation, mitochondrial segmentation (Fig. 4B-D) requires around 60 s per single-cell volumetric frame. Performing the analyses on such a large 4D dataset takes around 500 h, equivalent to nearly 21 days or 3 weeks, causing extreme delay in data analyses (Fig. 1D). However, with the advent of our optimized GPU-accelerated implementation, the run time of mitochondrial segmentation can be reduced by nearly 25-fold and the entire dataset can be processed in mere 20 h, making it much more practical. This new implementation marks a critical development in making MitoGraph a scalable and practical tool for modern 4D cell biology.

## Discussion

In this study, we present MitoGraph-GPU, a Python-based implementation that significantly lowers the computational barrier for 3D and 4D mitochondrial morphology analysis. Fast 4D imaging modalities like lattice light-sheet microscopy generate massive datasets where traditional CPU-based processing creates a severe bottleneck. By optimizing dominant filtering operations for the GPU, we achieved a ~ 30-fold reduction in runtime compared to the original CPU version. An advantage of this new implementation is its integration into the modern scientific Python ecosystem. While the original C + + version of MitoGraph was highly efficient, its reliance on specific libraries often limited native support primarily to macOS and Windows. By rebuilding the tool in Python, we provide native, cross-platform support for Windows, macOS, and Linux. For an organoid dataset requiring the processing of ~ 30,000 single-cell crops, MitoGraph-GPU reduces the total compute time from 500 h (CPU) to ~ 20 h (GPU). This effectively transforms a multi-week batch job into an overnight task on a standard lab workstation. Furthermore, our validation confirms that this acceleration maintains high segmentation fidelity, and yield almost identical biological readouts. Critically, our benchmarks were performed on consumer-grade, mid-range GPU hardware, demonstrating that massive mitochondria data processing is attainable without the need for high-performance computing clusters, which can be inaccessible to some research labs. Finally, MitoGraph-GPU provides standardized surface and skeleton outputs that serves as an upstream module for tracking tools like MitoTNT and related downstream workflows, enabling end-to-end analysis from 4D segmentation to graph-feature extraction, temporal remodeling metrics, and population-level phenotyping.

## Conclusion

MitoGraph-GPU is a GPU-accelerated implementation of the widely adopted mitochondrial segmentation tool MitoGraph, designed to handle the massive volumes of data generated by modern imaging techniques like lattice light-sheet microscopy. By effectively parallelizing the most computationally intensive filtering operations, the tool achieves a throughput increase of up to 30-fold while maintaining ~99.9% segmentation fidelity compared to the original CPU implementation. Our validation across diverse biological datasets confirms that the software produces accurate mitochondrial surfaces and skeletons with minimal variation in downstream morphological measurements. Most importantly, by reducing multi-week processing cycles into approximately 20-hour computations, this implementation enables the scalable analysis of 4D mitochondrial phenotyping and provides a practical pathway for researchers to explore complex organelle dynamics in high-throughput studies.

### Availability and requirements.

- Project name: MitoGraph-GPU 1.0.
- Project home page: https://github.com/schoeneberglab/MitoGraph-GPU (can be accessed through links on the original MitoGraph repository https://github.com/vianamp/MitoGraph).
- Operating system(s): Linux, Win, MacOS.
- Programming language: Python.
- License: GPL-2.0.

## Supplementary Information

Below is the link to the electronic supplementary material.

Supplementary Material 1

## Data Availability

The datasets generated and analyzed during the current study, including the 3D raw and processed imaging data of lung organoids acquired via lattice light-sheet microscopy, are currently not publicly available in a central repository due to their exceptionally large file size (exceeding several terabytes). However, these datasets are available from the corresponding author on reasonable request.

## Abbreviations

3D: Three-dimensional
4D: Four-dimensional
LLSM: Lattice Light-Sheet Microscopy
CPU: Central Processing Unit
GPU: Graphics Processing Unit
FFT: Fast Fourier Transform
GFP: Green Fluorescent Protein
RFP: Red Fluorescent Protein
