# Supplementary material for: GPU-accelerated MitoGraph for high-throughput three-dimensional mitochondrial morphology analysis
Source: BMC Bioinformatics. 2026 Jun 20;27:206. doi: 10.1186/s12859-026-06441-z (PMC13540902; doi:10.1186/s12859-026-06441-z)
Supplement: Supplementary file 1 [file 12859_2026_6441_MOESM1_ESM.docx]

GPU-accelerated MitoGraph for high-throughput three-dimensional

mitochondrial morphology analysis

Siddharth Nahar^1,2^, Zichen Wang^1,2^, Eric Arkfeld^1,2^, Gillian McMahon^1,2^, Hiroyuki Hakozaki^1,2^, Johannes Schöneberg^1,2^*

^1^ Department of Pharmacology, University of California, San Diego, San Diego, CA, 92093

^2^ Department of Chemistry and Biochemistry, University of California, San Diego, San Diego, CA 92093

* Address correspondence to

Johannes Schöneberg, Ph.D.

Departments of Pharmacology and Chemistry and Biochemistry

University of California, San Diego

Center for Neural Circuits and Behavior, Room 106B, La Jolla, CA 92093

Tel: 858-246-4755

Email: jschoeneberg@health.ucsd.edu

**Supplementary Figure 1. Log-log plot of runtime as a function of input data size.** Runtime benchmarks on a large volume built from stacking lung cell volumes show that both CPU (blue) and GPU (red) versions on a log-log axis. The best-fit lines are shown as dashed lines. Both implementations have slope=1, suggesting linear runtime. However, the GPU-accelerated version consistently achieves a ~30-fold speedup over the CPU baseline in this benchmark dataset.

**Supplementary Figure 2. Runtime benchmark for the original C++ MitoGraph and MitoGraph with vectorization by NumPy.** Despite the 6-fold acceleration through NumPy vectorization, the performance is still around 5-fold slower than the GPU-accelerated MitoGraph in main Figure 1D.
